# Supplementary material for: A network dynamical simulation model for the study of antibiotic resistance in microbial communities
Source: Gut Microbes. 2026 Sep 19;18(1):2734703. doi: 10.1080/19490976.2026.2734703 (PMC13596919; doi:10.1080/19490976.2026.2734703)
Supplement: Supplementary file 1 [file KGMI_A_2734703_SM9053.zip › supplementary_file_1/Figures/ba_delta_gc_frac.pdf]

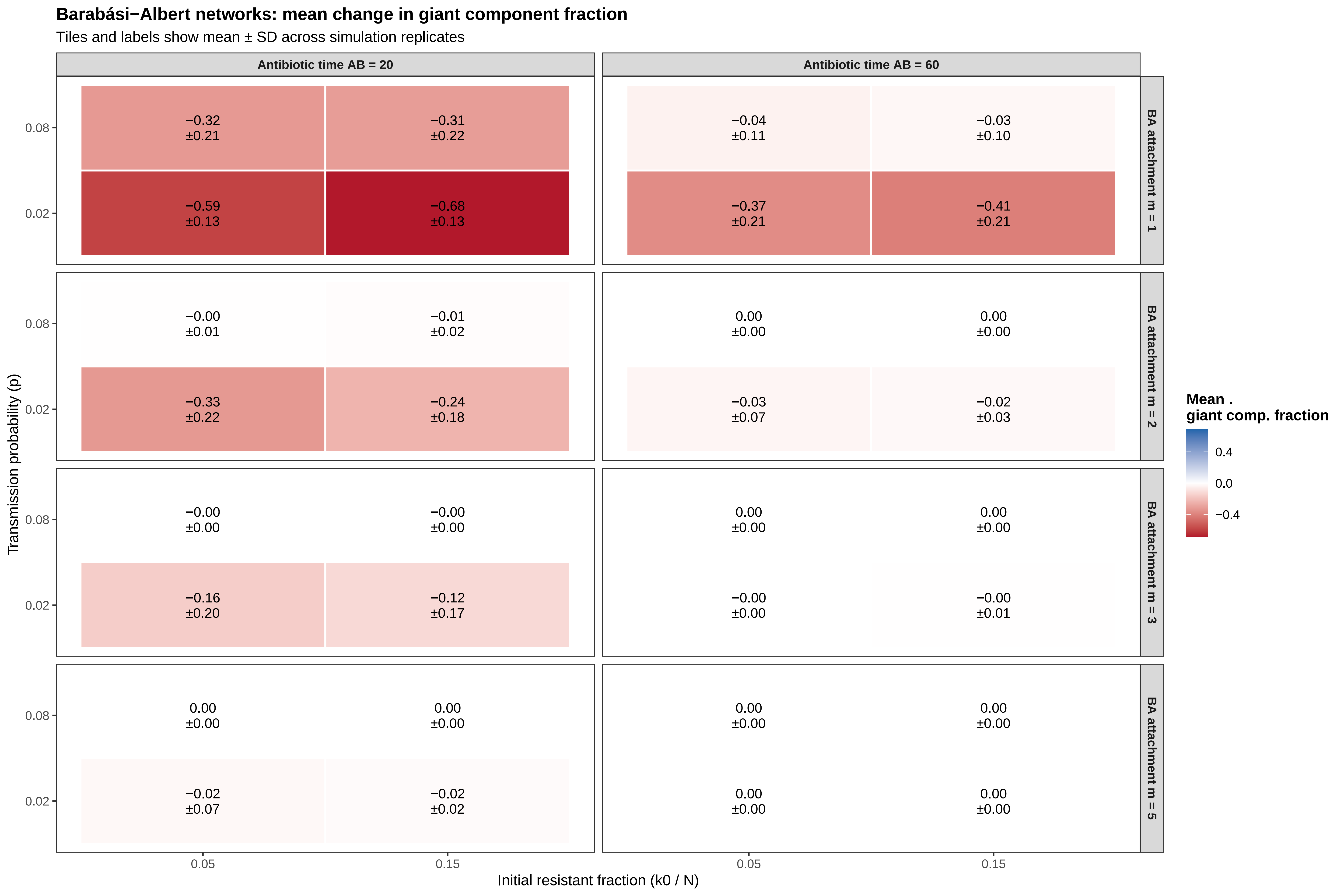
Barabási−Albert networks: mean change in giant component fraction
Tiles and labels show mean ± SD across simulation replicates
Antibiotic time AB = 20
Antibiotic time AB = 60
BA attachment m = 1
BA attachment m = 2
BA attachment m = 3
BA attachment m = 5
−0.32 ±0.21
−0.31 ±0.22
−0.04 ±0.11
−0.03 ±0.10
−0.59 ±0.13
−0.68 ±0.13
−0.37 ±0.21
−0.41 ±0.21
−0.00 ±0.01
−0.01 ±0.02
0.00 ±0.00
0.00 ±0.00
−0.33 ±0.22
−0.24 ±0.18
−0.03 ±0.07
−0.02 ±0.03
−0.00 ±0.00
−0.00 ±0.00
0.00 ±0.00
0.00 ±0.00
−0.16 ±0.20
−0.12 ±0.17
−0.00 ±0.00
−0.00 ±0.01
0.00 ±0.00
0.00 ±0.00
0.00 ±0.00
0.00 ±0.00
−0.02 ±0.07
−0.02 ±0.02
0.00 ±0.00
0.00 ±0.00
Transmission probability (p)
0.08
0.02
Initial resistant fraction (k0 / N)
0.05
0.15
Mean .
giant comp. fraction
0.4
0.0
−0.4
